# Convalescent plasma in the treatment of moderate to severe COVID-19 pneumonia: a randomized controlled trial (PROTECT-Patient Trial)

**DOI:** 10.1038/s41598-022-06221-8

**Published:** 2022-02-15

**Authors:** Karin van den Berg, Tanya Nadia Glatt, Marion Vermeulen, Francesca Little, Ronel Swanevelder, Claire Barrett, Richard Court, Marise Bremer, Cynthia Nyoni, Avril Swarts, Cordelia Mmenu, Thomas Crede, Gerdien Kritzinger, Jonathan Naude, Patryk Szymanski, James Cowley, Thandeka Moyo-Gwete, Penny L Moore, John Black, Jaimendra Singh, Jinal N. Bhiman, Prinita Baijnath, Priyesh Mody, Jacques Malherbe, Samantha Potgieter, Cloete van Vuuren, Shaun Maasdorp, Robert J Wilkinson, Vernon J Louw, Sean Wasserman

**Affiliations:** 1 Translational Research Department, Medical Division, South African National Blood Service, Roodepoort, South Africa; 2 Division of Clinical Haematology, Department of Medicine, Faculty of Health Sciences, University of Cape Town, Observatory, South Africa; 3 Division of Clinical Haematology, Departments of Internal Medicine, University of the Free State, Bloemfontein, South Africa; 4 Operations Testing Department, Operations Division, South African National Blood Service, Roodepoort, South Africa; 5 Department of Statistical Sciences, University of Cape Town, South Africa; 6 School of Clinical Medicine, University of the Free State, Bloemfontein, South Africa; 7 Division of Clinical Pharmacology, Department of Medicine, University of Cape Town, Observatory, South Africa; 8 Wellcome Centre for Infectious Diseases Research in Africa, Institute of Infectious Disease and Molecular Medicine, University of Cape Town, Observatory , South Africa; 9 Mitchells Plain Hospital and the University of Cape Town’ s Department of Medicine, Faculty of Health Sciences, University of Cape Town, Observatory, South Africa; 10 Processing Department, Operations Division, South African National Blood Service, Roodepoort, South Africa; 11 National Institute for Communicable Diseases of the National Health Laboratory Service, Johannesburg, South Africa and MRC Antibody Immunity Research Unit, School of Pathology, Faculty of Health Sciences, University of the Witwatersrand, Johannesburg, South Africa; 12 Department of Medicine, Walter Sisulu University, Livingstone Hospital, Gqeberha; 13 Capital Haematology Hospital and Bone Marrow Transplant Unit, Durban, South Africa; 14 Centre for Respiratory Diseases and Meningitis (CRDM), National Institute for Communicable Diseases, Johannesburg, South Africa Department of Virology, School of Pathology, Faculty of Health Sciences, University of the Witwatersrand, Johannesburg, South Africa; 15 Life Westville Hospital, Westville, South Africa; 16 Division of Infectious Diseases, Department of Internal Medicine, University of the Free State, Bloemfontein, South Africa; 17 3 Military Hospital and Department of Internal Medicine, University of the Free State, Bloemfontein, South Africa; 18 Pulmonology and Critical Care, Universitas Academic Hospital and Faculty of Health Sciences, University of the Free State; 19 Francis Crick Institute London NW1 1AT, UK; 20 Department of Infectious Diseases, Imperial College London, W12 0NN, UK; 21 Division of Infectious Diseases and HIV Medicine, Department of Medicine, Groote Schuur Hospital and University of Cape Town, Observatory, South Africa

**Keywords:** SARS-CoV-2, COVID-19, Convalescent plasma, HIV

## Abstract

**Background:**

There is a need for effective therapy for COVID-19 pneumonia. Convalescent plasma has antiviral activity and early observational studies suggested benefit in reducing COVID-19 severity. We investigated the safety and efficacy of convalescent plasma in hospitalized patients with COVID-19 in a population with a high HIV prevalence and where few therapeutic options were available.

**Methods:**

We performed a double-blinded, multicenter, randomized controlled trial in one private and three public sector hospitals in South Africa. Adult participants with COVID-19 pneumonia requiring non-invasive oxygen were randomized 1:1 to receive a single transfusion of 200mL of either convalescent plasma or 0.9% saline solution. The primary outcome measure was hospital discharge and/or improvement of ≥ 2 points on the World Health Organisation Blueprint Ordinal Scale for Clinical Improvement by day 28 of enrolment. The trial was stopped early for futility by the Data and Safety Monitoring Board.

**Results:**

103 participants, including 21 HIV positive individuals, were randomized at the time of premature trial termination: 52 in the convalescent plasma and 51 in the placebo group. The primary outcome occurred in 31 participants in the convalescent plasma group and and 32 participants in the placebo group (relative risk 1.03 (95% CI, 0.77 to 1.38). Two grade 1 transfusion-related adverse events occurred. Participants who improved clinically received convalescent plasma with a higher median anti-SARS-CoV-2 neutralizing antibody titre compared with those who did not (298 versus 205 AU/mL).

**Conclusions:**

Our study contributes additional evidence for recommendations against the use of convalescent plasma for COVID-19 pneumonia. Safety and feasibility in this population supports future investigation for other indications.

## Introduction

There are limited drugs with convincing evidence of efficacy for hospitalized patients with COVID-19. Host-directed therapies including dexamethasone [1], tocilizumab [2, 3], and baricitinib [4], and the monoclonal antibody casirivimab/imdevimab cocktail [5], and anakinra [43] are associated with survival benefit. Remdesivir, an antiviral agent, has modest impact on shortening the time to recovery. [6] Of these, only dexamethasone is widely available in resource-limited settings and there is an urgent need for accessible therapeutic options for COVID-19 pneumonia.

Transfusion with convalescent plasma (CP) provides antiviral activity through transfer of neutralizing antibodies and possibly other immune components. [7] Observational studies suggested CP therapy may improve outcomes in severe viral infections, [8] including lower mortality and earlier hospital discharge in SARS-CoV. [9] There have been no safety signals associated with CP use in the treatment of severe viral infections. [10] CP is therefore an attractive potential therapy for COVID-19, particularly in resource-limited settings where access to other novel drugs is limited, [11] given its potential for rapid and relative low cost local production. [12]

In the absence of other therapeutic options, COVID-19 convalescent plasma (CCP) was widely deployed outside of clinical trials in the first year of the pandemic. Early experience from uncontrolled observational studies suggested efficacy for CCP, including improved survival, decreased viral load, and radiological improvement. [13, 14] There was a dose-response relationship in an analysis of a large expanded access programme, with reduced 30-day mortality among patients receiving CCP with higher-titre anti-SARS-CoV-2 spike IgG compared with those who received lower titres, providing biological plausibility for clinical efficacy. [15] Encouraging results were also observed in a small randomised control trial (RCT) in India where CCP was associated with improvement in respiratory parameters and a shortened recovery time. [16] Another RCT from New York and Brazil found significantly improved survival at day 28 in participants treated with CCP. [17] By contrast, the RECOVERY trial, a large RCT with clinical endpoints, was halted prematurely as high titre CCP did not improve survival in hospitalized patients with COVID-19 in the UK. [18]

Prior to the announcement of the RECOVERY trial results and in the context of rapidly emerging observational data supporting potential efficacy and safety of CCP, we undertook a randomized controlled trial to test whether CCP improved clinical recovery for COVID-19 pneumonia in an African population with high HIV prevalence and limited available treatment options.

## Methods

### Study design and population

The PROTECT-Patient trial, A **PRO**spective, randomized, placebo-controlled, double-blinded phase III clinical trial of the **T**herapeutic use of conval**E**s**C**en**T** plasma in the treatment of patients with moderate to severe COVID-19) evaluated the efficacy and safety of CCP for hospitalized patients with COVID-19 pneumonia. The trial took place at one private sector and three public sector hospitals in South Africa. The trial was first registered on 24/04/2020. The trial was sponsored by the South African National Blood Service (SANBS) and was conducted in accordance with the principles of the International Conference on Harmonisation–Good Clinical Practice guidelines, approved by the South African Health Products Regulatory Authority and the Human Research Ethics Committees of SANBS (2019/0524, 24/04/2020), University of Cape Town (312/2020, 14/07/2020), University of the Free State (UFS2020/1253/2710, 22/09/2020), University of Walter Sizulu (086/2020, 04/11/2020) and the Life Hospital group (07052020/1 07/07/2020). The trial is registered on clinicaltrials.gov (NCT04516811 (https://clinicaltrials.gov/ct2/show/NCT04516811), 18/08/2020) and the protocol is available as part of supplementary material.

Informed consent was obtained from hospitalized patients ≥ 18 years of age were eligible for inclusion if they had laboratory confirmation of SARS-CoV-2 infection by reverse transcriptase polymerase chain reaction (RT-PCR) on any respiratory sample, radiologic evidence of pneumonia with pulmonary infiltrates on chest x-ray, oxygen saturation (SpO2) of < 94% on room air and requiring non-invasive oxygen therapy. We excluded patients who were mechanically ventilated or where survival for < 24 hours was expected. Co-enrolment into another investigational therapy trial was not permitted.

### Randomization, blinding, and intervention

Eligible and consenting participants were randomized (1:1) to receive either a single infusion of 200-250 mL CCP or 200 mL of placebo (0.9% normal saline), together with local standard of care. Random assignment was stratified by study site, age (≥or < 65 years), and body mass index (BMI) (≥or < 30kg/m^2^). An electronic randomisation application (REDCap) hosted by SANBS [19], was used to generate the treatment allocation. To mask treatment allocation, investigational product (IP) was covered in opaque paper wrapping prior to dispatch from the blood bank. Participants were transfused within 24 hours of randomization. CCP or placebo was administered over 20-30 minutes; vital signs were monitored at 15-minute intervals for one-hour post initiation of transfusion. Use of other treatments, including corticosteroids and anticoagulation, was at the discretion of treating clinicians; remdesivir was unavailable for routine use.

CCP was collected by SANBS and the Western Cape Blood Service (WCBS) from donors who had recovered from SARS-CoV-2 infection, confirmed by positive nasopharyngeal swab RT-PCR (Supplementary material). SARS-CoV-2 antibody titre testing was performed by the National Institure of Communicable Diseases (NICD) using an in-house enzyme-linked immunosorbent assay (ELISA) assay based on the assay developed by Mount Sinai Hospital, [20] which detected the presence of anti-spike and -receptor binding domain antibodies. Testing for neutralizing antibodies (nAb) was performed on stored samples once the assay was developed and validated (methods described elsewhere). [21] Initially we selected donor units with an anti-spike IgG optical density (OD_450nm_) of ≥ 0.4, which was considered to be positive. [20] As more information emerged regarding the importance of nAb titers, we aimed to transfuse CCP with nAb titres of 1:160 or higher based on findings from other clinical trials [22]. However, in line with Food and Drug Administration (FDA) guidance [23] on the use of anti-spike binding antibody as a proxy for nAb, we moved to using CCP with anti-spike protein IgG OD_450nm_ values greater than 2.0, which correlated well with nAb titres ≥ 1:160. [20]

### Clinical and laboratory monitoring

We performed daily in-person or medical record assessments for adverse events, clinical status, and oxygen utilization during hospital admission. For participants discharged prior to day 28, clinical status and adverse events were determined via telephone. Concomitant medication was recorded at each clinical visit. Phlebotomy for safety and inflammatory markers was performed on the day of transfusion and when possible, on days 2, 5, 10, 14 and 28 during hospitalisation. A nasopharyngeal swab for SARS-CoV-2 genomic sequencing was collected at enrolment in a subset of patients and sent to the NICD for RT-PCR testing using the 3 Allplex™ 2019-nCoV assay (Seegene Inc). Retained RNA samples were processed using a commercially available version of the SARS CoV-2 ARTIC library preparation protocol (NEBNext ARTIC SARS-CoV-2 FS Library Prep Kit, cat# E7658 (Illumina, San Diego, California, USA)). Following library preparation, samples were sequenced on the NextSeq 500 platform (Illumina, San Diego, California, USA). A minimum of 1 million 2x150 bp PE reads were generated per sample. Analysis of sequence data was performed by the NICD. Whole genome assembly was conducted using the Exatype SARS-CoV-2 platform (https://sars-cov-2.exatype.com/).

### Outcomes

The primary outcome measure was successful treatment at Day 28 post-randomization, defined as acute care hospital discharge or clinical improvement of ≥ 2 points on an ordinal scale recommended by the World Health Organization [24]: 0 -uninfected; 1 -ambulatory with no limitation of activites; 2 -ambulatory with limitation of activities; 3 -hospitalized, not requiring supplemental oxygen; 4 -hospitalized, requiring supplemental oxygen; 5 -hospitalized, requiring high-flow oxygen therapy or non-invasive mechanical ventilation; 6 - hospitalized, requiring extracorporeal membrane oxygenation, invasive mechanical ventilation (IMV), or both; 7 -ventilation and additional organ support; 8, death. Secondary efficacy outcomes included clinical improvement and hospital discharge as separate categories; survival at Day 28 post-randomization; invasive mechanical ventilation (IMV); duration of hospitalisation; and time from randomization to clinical improvement and death. Safety outcomes were adverse events of special interest (transfusion associated circulatory overload, transfusion associated acute lung injury, allergic transfusion reactions); and serious and Grade 3 or 4 adverse events.

### Sample size

We hypothesized that a single transfusion of CCP would be associated with improved treatment success compared with saline placebo in patients hospitalized with COVID-19 pneumonia. Based on observational studies at the time of protocol development, [25–27] we assumed that approximately 30% of patients with COVID-19 pneumonia would not reach the primary endpoint event of clinical improvement. We estimated a sample size of 600 participants (300 per treatment group) would provide 80% power at 2-sided alpha to detect a 33% difference in relative risk (10% absolute difference) in the primary outcome between the two arms.

### Early trial termination and analysis

The trial management committee, in discussion with the trial DSMB, paused recruitment on 14 January 2021 in response to evidence that convalescent plasma, collected from people infected with ‘wild-type’ virus during the first wave in South Africa, had poor neutralizing activity against the Beta (B.1.351/501Y.V2) SARS-CoV-2 variant presumed to have become the dominant variant in trial participants. [28] The trial DSMB requested an unplanned interim analysis with futility calculations based on conditional power. The 103 participants who received IP at the time of pausing provided an information fraction of 0.17 with observed event probabilities of 0.63 and 0.61. Conditional power for a significance difference at the end of the trial based on the observed trend was 40%; if no effect was assumed from the time recruitment was paused to the end of the trial, conditional power was 3%. Based on these low conditional probabilities, and emerging data from the RECOVERY trial, the DSMB recommended that the trial be stopped for futility on 10 February 2021. To report findings, prevalence of endpoints in the intention to treat population were estimated at day 28 and/or date of discharge using risk ratios and 95% confidence intervals. Due to lack of power as a result of early trial cessation, no formal statistical comparisons were done. We used Kaplan-Meier estimates to compare and illustrate time to clinical improvement and death in the treatment groups. We used STATA V.17 (STATACORP, Texas) to perform the statistical analysis.

## Results

**Figure 1:**
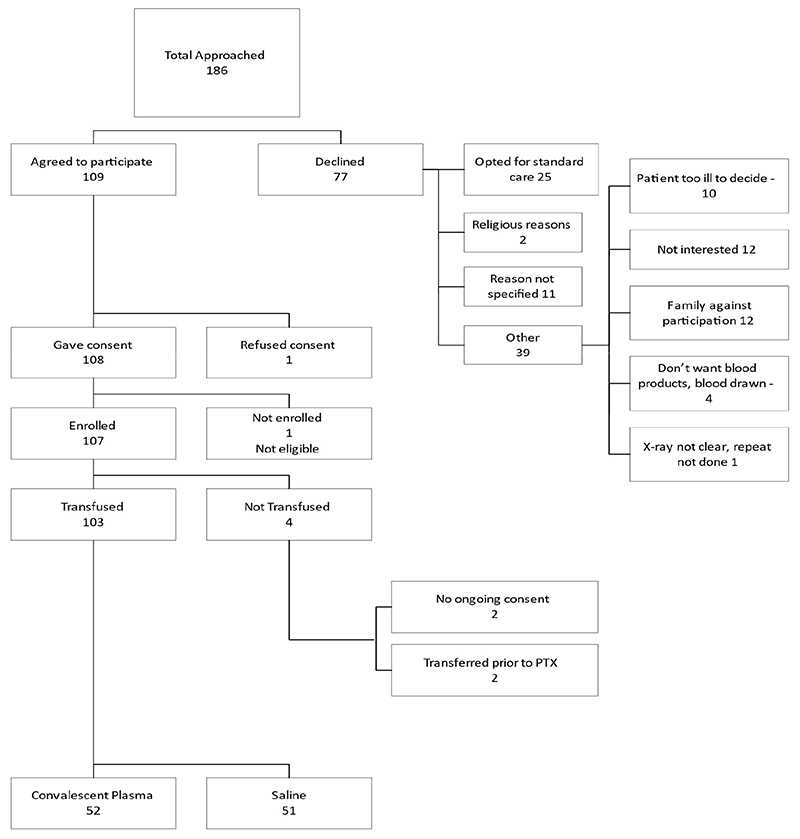
Consort diagram illustrating numbers of participants with moderate-severe COVID-19 randomized (1:1) to treatment with convalescent plasma or placebo

107 participants were enrolled between 30 September 2020 and 14 Jan 2021; 103 were transfused with IP (two participants withdrew consent and two were transferred to alternative facilities prior to transfusion): 52 participants received CCP and 51 were given placebo (Figure 1).

The trial groups were well balanced in terms of baseline characteristics, disease severity, and routine management (Tables 1 and 2). Twenty-one (25%) of those with known HIV status (n = 84) were HIV positive, 16 (76%) of whom were on antiretroviral therapy (ART). Median CD4 count for the HIV positive participants was 596 (interquartile range (IQR) 242-1029). Co-morbidities, including a high prevalence of diabetes and hypertension, were present in 42 (80.8%) participants. More than half of participants (n = 56, 54.4%) were classified as obese. [29] The median time from symptom onset to IP infusion was 9 (IQR 6-11) days. Corticosteroids were used in 97 (94.2%); 98 (95.1%) participants were prescribed heparin prophylaxis, mostly with therapeutic doses.

**Figure 2:**
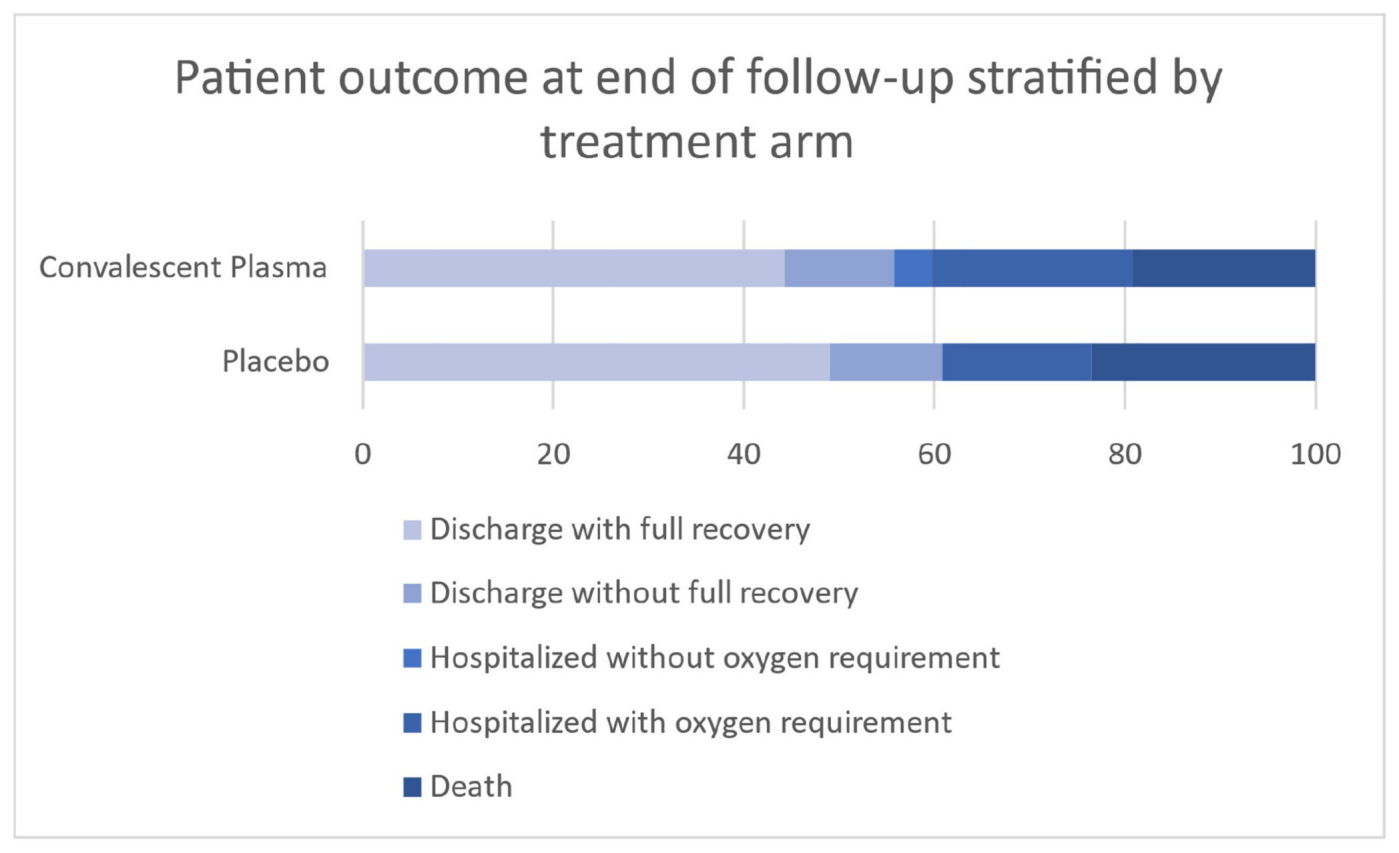
Proportion of clinical outcomes for 103 participants with moderate-severe COVID-19 randomized to treatment with convalescent plasma versus placebo at day 28 post recruitment

**Figure 3:**
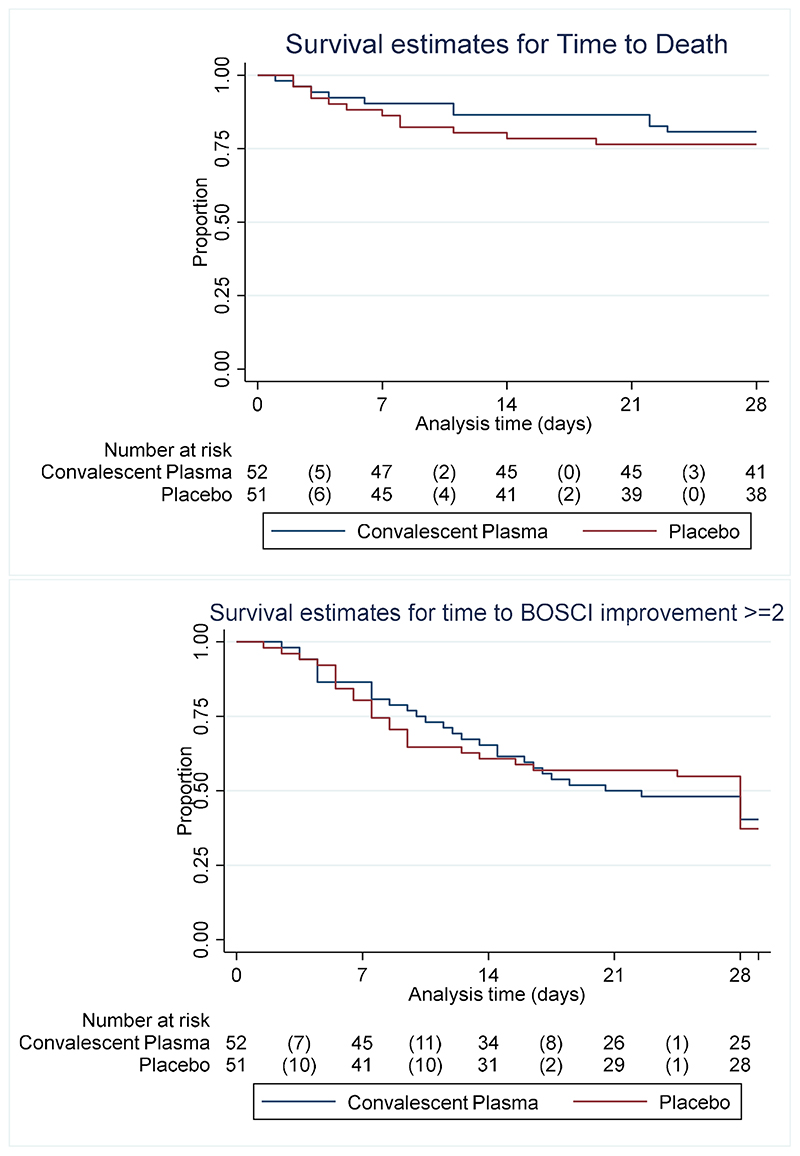
Kaplan-Meier survival analysis of time to death and separately for improvement by >= 2 BOSCI points in 103 participants with moderate-severe COVID-19 randomized treatment with convalescent plasma versus placebo *BOSCI: World Health Organization Blueprint Ordinal Scale for Clinical Improvement

Information on the primary endpoint was unavailable for six participants who were uncontactable after initial discharge or transfer, however sensitivity analysis did not change the findings. The primary outcome was therefore assessed in 97/103 (94.2%) participants: 31 (66.0%) in the CCP group and 32 (64.0%) in the placebo group experienced ≥2 point BOSCI improvement or discharge by Day 28, relative risk 1.03 (95% CI, 0.77 to 1.38) (Table 3). All participants who were discharged demonstrated a clinical improvement (BOSCI improvement ≥ 2) (Figure 2). There were 11 and 13 deaths in the CCP and placebo groups, respectively. Four participants required invasive mechanical ventilation, one in the CCP group and three in the placebo group. There was no difference in time to death or clinical improvement between treatment groups (Figure 3). Among HIV positive participants, the primary outcome was achieved in two (33%) and nine (60%) participants in the CCP and placebo groups, respectively.

The total number of adverse events, including serious adverse events, was similar across treatment groups (Table 4). Two transfusion-related adverse events occurred, both grade 1 allergic reactions. Six grade 3 or higher adverse events were recorded among HIV-positive participants who were transfused CCP, all assessed as unrelated to the study drug.

**Figure 4:**
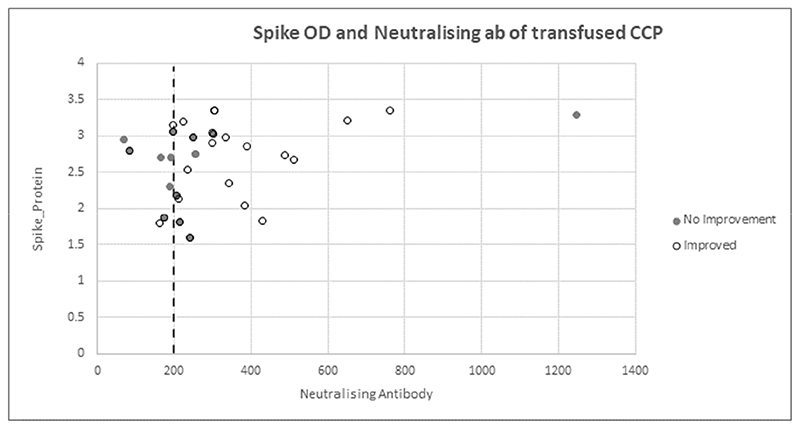
Correlation of neutralizing antibody titer and spike optical density, partitioned by clinical improvement status in patients who received CCP.

In the transfused CCP, the median anti-spike protein IgG optical density (OD_450nm_) was 2.7 AU/mL (IQR, 2.0 to 3.0); and the median anti-SARS-CoV-2 neutralizing antibody titre was an inhibitory dilution at which 50% neutralization is attained (ID_50_) of 1:234 AU/mL (IQR, 194 to 304; range, 71 to 1245). Participants who demonstrated clinical improvement received CCP transfusions with higher median anti-SARS-CoV-2 neutralizing antibody titres compared with those who did not : ID_50_ of 1:298 (IQR: 212 – 374) versus 1:205 (IQR: 181 – 254) (Figure 4). Of those who showed clinical improvement, 21 (81%) had an antibody titre > 1:200 ID_50_ (Figure 4).

SARS-CoV-2 genomic sequencing was performed on available baseline nasopharyngeal samples from 66 participants. SARS-CoV-2 variants were similarly distributed across treatment groups. The beta variant was detected in 45 (68.2%) samples. In the CCP group, 6/22 (27.7%) participants with the beta variant died and 1/10 (10.0%) participants with other SARS-CoV-2 variants died. Clinical improvement by day 28 occurred in 11/18 (61.1%) of participants with beta variant infection who received CCP, and in 7/10 (70.0%) of those infected with other variants.

## Discussion

Our trial showed that the use of therapeutic CCP for hospitalized patients with COVID-19 pneumonia was not associated with clinical improvement in a South African population with a high HIV-prevalence. Although our final sample size did not support formal hypothesis testing, the lack of any signal of benefit led to a conclusion of futility, and is consistent with findings from randomized controlled trials in other settings[SW1] [18, 22, 30]. Notwithstanding early trial termination, our study contributes additional evidence for recommendations against use of CCP for established COVID-19 pneumonia, especially in a high HIV-prevalence setting, and may provide some insights into reasons for the lack of clinical efficacy.

Observational studies suggest that the clinical effect of CCP is related to neutralizing antibody titre [15]. There has been variability and lack of standardisation in transfused neutralizing titres across trials, which may explain some of the heterogeneity in outcomes with CCP. It is an operational challenge to perform neutralisation assays in real time, and similarly to other studies, we selected CCP units on anti-spike IgG antibodies that correlated with neutralizing titres. Consequently, we used a wide dosing range in transfused CCP in our trial, highlighting the challenge of CCP standardisation in future studies, or if implementation is contemplated. Informative subgroup analyses were not possible with our limited sample size, but we did observe higher neutralizing titres among participants with better outcomes. However, given the lack of clinical effect in the RECOVERY trial, which only transfused high anti-spike titre CCP, it is unlikely that selecting donor plasma with titres in accordance with current FDA guidelines, [23], would have altered our main finding.

The majority of CCP donations for the PROTECT trial were collected during the first COVID-19 wave in South Africa, during which donors were likely infected with the ‘wild type’ SARS-CoV-2. Due to delays in regulatory approvals, recruitment was unable to commence until the beginning of the second wave when the beta variant had become the predominant circulating SARS-CoV-2 strain, [31]. This was subsequently confirmed by sequencing in over two-thirds of our participants with available samples. *In vitro* experiments indicated that SARS-CoV-2 antibodies in convalescent plasma provided for the PROTECT trial, obtained from donors infected before the emergence of beta, was significantly less effective at neutralizing pseudovirus expressing beta variant spike protein [28]. SARS-CoV-2 variants have also shown resistance to neutralisation by anti-receptor binding domain monoclonal antibodies [32], and antigenic variability of SARS-CoV-2 with emergence of new variants will be a major obstacle to deployment of these therapeutic strategies. Although CCP may contribute some indirect antiviral effect through non-neutralizing antibody activity [7], which is preserved in convalescent plasma against multiple variants [28], this appears unlikely to translate into clinical efficacy.

Clinical studies suggest CCP [15, 33, 34] and monoclonal antibody [35] therapy may provide benefit early in the course of COVID-19 when SARS-CoV-2 viral load is highest at around 3 days post-diagnosis [36], but this has been inconsistent [37]. The median time from symptom onset to transfusion in our trial was nine days, which is comparable to other negative trials of CCP for inpatients with COVID-19 pneumonia [17, 18, 41, 42]. The RECOVERY trial did not find benefit for earlier CCP administration on stratified analysis, and a trial evaluating monoclonal antibody therapy was stopped early for failing to demonstrate efficacy among hospitalized patients, even with low oxygen requirements [38], suggesting that antibody based therapies are unlikely to be effective after activation of excessive host inflammation associated with severe disease.

CCP may have therapeutic potential in other contexts and patient groups and more likely as a prophylactic for severe COVID-19 in individuals with comorbidities. A randomized controlled trial showed that transfusion of CCP within three days of symptom onset reduced progression to severe COVID-19 among older people in Argentina [33], and there are at least thirteen active trials evaluating CCP for treatment and prophylaxis of mild or moderate COVID-19 (https://covid-nma.com/dataviz). There is also accumulating evidence from case reports of good outcomes with CCP use in a heterogeneous group of patients with primary and secondary immunodeficiency, including those with haematological malignancy and solid organ transplantation [39]. HIV may be a risk factor for worse outcomes with COVID-19 [40], but data is scarce on the use of CCP in this patient group. Our trial included 21 HIV-positive participants, six of whom received CCP. Although this small number precludes definitive conclusions, absence of a major safety signal is somewhat reassuring. In line with experience in other settings [10], CCP use was safe in our overall cohort, supporting future evaluation for different indications in high HIV burden populations.

The premature termination of the PROTECT trial illustrates the complexity of undertaking clinical research in a rapidly evolving global pandemic. Inconsistent case numbers, viral evolution, and rapidly changing evidence during the trial period contributed to this challenge. Despite this, our trial demonstrated the feasibility of deploying CCP in a resource-limited setting and contributed knowledge on the use of this therapeutic strategy for COVID-19 pneumonia. Our experience highlights the necessity of globally networked clinical trial sites and harmonised study protocols to more efficiently evaluate interventions among diverse populations during a pandemic.

## Supplementary Material

Supplementary text

## Figures and Tables

**Table 1:** Participant characteristics at baseline in 103 participants with severe COVID-19 randomized to treatment with convalescent plasma versus placebo

	CCP, n (%)	Placebo, n (%)	Total (%)
**TOTAL**	52	51	103
**Age Group (years)**			
40	9 (17.3)	6 (11.8)	15 (14.5)
40-60	26 (50.0)	26 (51.0)	52 (50.5)
>60	17 (32.7)	19 (37.3)	36 (35.0)
Age- median (IQR)	54 (46-62)	57 (47-64)	56 (46-63)
**Gender**			
Female	31 (59.6)	30 (58.8)	61 (59.2)
Male	21 (40.4)	21 (41.2)	42 (40.8)
**HIV status**			
Positive	6 (11.5)	15 (28.8)	21 (18.5)
on ART	5 (83.3)	11 (73.3)	16 (76.2)
not on ART	1 (16.7)	4 (26.7)	5 (24.8)
Negative	37 (71.2)	26 (51.0)	63 (62.1)
Unknown	9 (17.3)	10 (19.6)	19 (19.4)
**Smoking**			
Current smoker	5 (9.6)	6 (11.8)	11 (10.7)
Ex-smoker	11 (21.2)	11 (21.6)	22 (21.7)
**BMI**			
BMI >= 30kg/m^2^	29 (55.8)	27 (52.9)	56 (54.4)
BMI -Median (IQR)	31.2 (26.8-37.6)	31.0 (26.8-36.0)	
**SARS-CoV-2 clade on sequencing**	34	32	66
19A	2 (5.9)	2 (6.3)	4 (6.1)
20A	4 (11.8)	6 (18.8)	10 (15.2)
20B	5 (14.7)	2 (6.3)	7 (10.6)
20H/501Y.V2	23 (67.6)	22 (68.8)	45 (68.2)

**Table 2:** Clinical and laboratory characteristics at baseline in 103 participants with severe COVID-19 randomized to treatment with convalescent plasma versus placebo

	CCP n (%)		Placebo n (%)		Total n (%)	
**TOTAL**	52 (50.5%)		51 (49.5%)		103 (100%)	
BOSCI* score at enrolment						
4	40 (76.9%)		42 (82.4%)		82 (79.6%)	
5	12 (23.1%)		9 (17.6%)		21 (20.4%)	
Co-morbidities**						
Chronic Kidney disease	2 (3.8%)		1 (2%)		3 (2.9%)	
Diabetes	25 (48.1%)		15 (29.4%)		40 (38.8%)	
Hypertension	28 (53.8%)		28 (54.9%)		56 (54.4%)	
Obesity	24 (46.2%)		25 (49%)		49 (47.6%)	
Cardiovascular disease	1 (1.9%)		2 (3.9%)		3 (2.9%)	
Cancer	0 (0%)		2 (3.9%)		2 (1.9%)	
Chronic pulmonary disease	2 (3.8%)		2 (3.9%)		4 (3.9%)	
**Concomitant Medication**						
Antibiotic	14 (26.9%)		11 (21.6%)		25 (24.3%)	
Enoxaparin sodium	48 (92.3%)		50 (98%)		98 (95.1%)	
• Therapeutic dose	38 (71.2%)		36 (72%)		(%)	
• Prophylactic dose	10 (20.8%)		14 (28%)		(%)	
Inotropes	1 (1.9%)		2 (3.9%)		3 (2.9%)	
Steroids						
(Dexamethasone/prednisone)	50 (96.2%)		47 (92.2%)		97 (94.2%)	
	**n**	**Median (IQR)**	**n**	**Median (IQR)**	**n**	**Median (IQR)**
**Average days from symptom onset to**						
Hospitalisation	52	5(3-7)	51	5 (3.5-9.5)	103	5 (3-8)
Enrolment	52	8 (6-10.25)	51	8 (6-11)	103	8 (6-11)
Transfusion	52	9 (6-11)	51	9 (7-12)	103	9 (6-11)
**Baseline clinical measurements**						
Pulse (beats/min)	52	85 (74-99)	50	87 (77-95)	102	86 (75-95)
Respiratory rate (breaths/min)	52	26 (22-30)	51	28 (22-32)	103	28 (22-32)
Lowest Sp02 prior to starting O2 (%)	50	85.5 (82-88)	51	86 (78-89)	101	86 (80-88)
PaO2 (kPa)	36	10.3 (8.0-14.0)	35	9.2 (6.0-13.9)	71	9.3 (7.1-14.0)
**Baseline laboratory measurements**						
Creatinine Clearance (mL/min)	43	86.6 (61.0-106.0)	45	90.2 (82.3-104.8)	88	88.9 (73.2-104.8)
Ferritin (μg/L)	46	454.5 (161-776)	45	495.0 (263-1177)	91	495 (213-883)
D-Dimer (μg/mL)	42	0.5 (0.3-1.7)	43	0.5 (0.3-1.2)	85	0.5 (0.3-1.4)
Haemoglobin (g/dL)	45	12.9 (11.5-13.9)	47	13.1 (12.1-14.6)	92	13.1 (11.8-14.0)
Absolute white cell count (x10^9^/L)	45	12.1 (10.4-15.5)	48	9.8 (8.3-14.2)	93	11.29 (8.4-14.7)
Absolute neutrophil count (x10^9^/L)	44	10.0 (6.9-12.8)	47	8.0 (6.3-11.1)	91	9.13 (6.74-12.72)
Platelets (x10^9^/L)	45	346 (261-423)	47	322 (229-401)	92	339 (257.5-405.5)
Alanine Aminotransferase (ALT) (IU/L)	41	30 (20-48)	43	35 (26-57)	84	31 (23-51)
C-reactive protein (μg/L)	44	135 (56-202)	43	111 (62-222)	87	122 (57-207)
Troponin-T (ng/ml)	41	7 (5-14)	45	9 (6-18)	86	9 (5-17)
Lactate dehydrogenase (LDH) (U/L)	37	393 (298-459)	34	456 (357-711)	71	415 (314-571)

* minimum BOSCI score of 4 was required for eligibility. BOSCI 4: hospitalized, mild disease, on oxygen by mask or nasal prongs. BOSCI 5: hospitalized, severe disease, non-invasive ventilation or high flow oxygen.

** comorbidities are listed as number of comorbidities

**Table 3:** Primary and secondary efficacy outcomes in 103 participants with severe COVID-19 randomized to treatment with convalescent plasma versus placebo

Primary outcome	CCP; n (%)	Placebo; n (%)	RR (95% CI)
Clinical improvement by D28	31/47 (66.0)	32/50 (64.0)	1.03 (0.77 to 1.38)
**Secondary efficacy outcomes**			
Discharge from hospital by D28	28/46 (60.9)	31/50 (62.0)	1.21 (0.84 to 1.74)
BOSCI improvement: >= 2 by D28	31/47 (66.0)	32/50 (64.0)	1.03 (0.77 to 1.38)
Death by D28	11/52 (21.5)	13/51 (25.5)	0.83 (0.41 to 1.68)
Invasive mechanical ventilation	1/52 (1.9)	3/51 (5.9)	0.33 (0.04 to 3.04)

Six participants were discharged from primary acute care prior to Day 28 but were lost to follow up as they were uncontactable at Day 28. Clinical improvement is a composite of discharge from hospital and/or improvement in BOSCI score by >=2 by Day 28.

**Table 4:** Number of adverse events in 103 participants with severe COVID-19 randomized to treatment with convalescent plasma versus placebo

Adverse Events	CCP	Placebo	TOTAL
Number of participants with adverse events	23	17	40
Number of Adverse Events	32	27	59
Number of Serious Adverse Events	18	18	36
Number of Deaths	11	13	24
Number of Transfusion related AE	1	1	2

## Summary

Convalescent plasma (CP) did not improve outcomes for COVID-19 pneumonia in a high HIV prevalence setting; however a favourable safety profile suggests CP could be explored for other indications.
