# Supplementary material for: Convalescent plasma in the treatment of moderate to severe COVID-19 pneumonia: a randomized controlled trial (PROTECT-Patient Trial)
Source: Sci Rep. Author manuscript; Available in PMC 2022 Feb 18. (PMC8847351; doi:10.1038/s41598-022-06221-8)
Supplement: Supplementary text [file EMS140931-supplement-Supplementary_text.docx]

**Convalescent plasma in the treatment of moderate to severe COVID-19 pneumonia: a randomized controlled trial (PROTECT-Patient Trial)**

**SUPPLEMENTARY TEXT**

Convalescent plasma (CCP) was collected from donors using the following criteria: confirmed previous SARS-CoV-2 infection with a minimum of ≥ 14 days post two sequential negative nasopharyngeal swab PCR tests performed ≥ 24 hours apart, or ≥ 28 days post last symptoms. All CCP donors met standard eligibility criteria for source plasma donors including: age ≥ 18 years, weight ≥ 55kg, and a healthy lifestyle to reduce the risk of transfusion-related infections. We limited donor recruitment to males and nulliparous females to minimize the risk of transfusion related acute lung injury. Standard nucleic acid amplification and serology tests for HIV-1, hepatitis B virus, and hepatitis C virus were performed in parallel at each donation. ABO, Rh Blood grouping and screening for non-ABO red blood cell antigens were also performed on all plasma donations. Approximately 650 mL of plasma were collected by apheresis at each donation; donors were encouraged to donate every two weeks. Each unit was aliquoted into two samples of 200 mL each and pathogen reduced using a riboflavin, [39] or amotosolen mediated process. [40]
